# Influence of the Sodium Titanate Crystal Size of Biomimetic Dental Implants on Osteoblastic Behavior: An In Vitro Study

**DOI:** 10.3390/biomimetics10010043

**Published:** 2025-01-12

**Authors:** Saray Fernández-Hernández, Javier Gil, Daniel Robles-Cantero, Esteban Pérez-Pevida, Mariano Herrero-Climent, Aritza Brizuela-Velasco

**Affiliations:** 1 Bioengineering Institute of Technology, Facultad de Medicina y Ciencias de la Salud, Universidad Internacional de Catalunya, C/Josep Trueta s/n, 08195 Sant Cugat del Vallés, Spain; sfernandezh@uemc.es; 2 DENS-ia Research Group, Faculty of Health Sciences, Miguel de Cervantes European University, C/del Padre Julio Chevalier 2, 47012 Valladolid, Spain; drobles@clinica.uemc.es (D.R.-C.); eperezpevida@gmail.com (E.P.-P.); 3 Bioinspired Oral Biomaterials and Interfaces, Department Ciencia e Ingeniería de Materiales, Escoal d’Enginyeria Barcelona Est, Universitat Politècnica de Catalunya, Av. Eduard Maristany 16, 08019 Barcelona, Spain; 4 Independent Researcher, Porto Dental Institute, Av. De Montevideo 810, 4150-518 Oporto, Portugal; dr.herrero@herrerocliment.com

**Keywords:** dental implants, titanium, sodium titanate, biomimetic surface, osteoblasts

## Abstract

Treating the surfaces of dental implants in an alkaline medium allows us to obtain microstructures of sodium titanate crystals that favor the appearance of apatite in the physiological environment, producing osteoconductive surfaces. In this research, 385 discs made of titanium used in dental implants underwent different NaOH treatments with a 6M concentration at 600 °C and cooling rates of 20, 50, 75, and 115 °C/h. Using high-resolution electron microscopy, the microstructures were observed, and the different crystal sizes were determined and compared with control samples (those without biomimetic treatment). Roughness, wettability, surface energy and the sodium content of the surface were determined. The different surfaces were cultured with human osteoblastic cells; cell adhesion was determined at 3 and 14 days, and the degree of mineralization was determined at 14 days via alkaline phosphatase levels. Variations in the microstructure and size of sodium titanate crystals in NaOH solutions rich (1 g/L) or low in calcium (approximately 100 ppm) were determined. The results show that as the cooling rate increases, the size of the crystals decreases (from 0.4 μm to 0.8 μm) except for the case of 115 °C/h, when the rate is too fast for crystalline nucleation to occur on the surface of the titanium. The thermochemical treatment does not influence the roughness or the cooling rate since a Sa of 0.21 μm is maintained. However, the presence of titanate causes a decrease in the contact angle from 70° to 42° and, in turn, causes an increase in the total surface energy from 35 to 49.5 mJ/m^2^, with the polar component standing out in this energy increase. No variations were observed in the thermochemical treatments in the presence of sodium, which was around 1200 ppm. It was observed that as the size of the crystals decreases, cell adhesion increases at 3 days and decreases at 14 days. This is because finer crystals on the surface are already in the mineralization process, as demonstrated using the level of alkaline phosphatase that is maximal for the cooling rate of 75 °C/h. It was possible to confirm that the variations in the concentrated NaOH solutions with different calcium contents did not affect the crystal sizes or the microstructure of the surface. This research makes it possible to obtain dental implants with different mineralization speeds depending on the cooling rate applied.

## 1. Introduction

Advances in surface characteristics have led to a more predictable and quicker osseointegration of dental implants which, in turn, has initiated a progressive change in loading protocols in recent years [1,2]. Immediate loading has been defined as the connection of the rehabilitation to the implant in occlusion with the opposing dentition earlier than 1 week after implant placement, while early loading is defined as occurring between 1 week and 2 months subsequent to implant placement [3]. Predictable results in terms of implant survival rate and marginal bone loss have been attained with immediate and early loading procedures in different clinical situations, such as in the treatment of either totally or partially edentulous patients with fixed prostheses [4,5,6]. In general, from a clinical point of view, immediate loading is more common in the rehabilitation of the anterior maxillary and mandibular sectors, mainly for esthetic reasons and the configuration of an adequate emergency profile. Nevertheless, in the rehabilitation of the premolar and molar areas of the maxilla and mandible with dental implants, the placement of fixed provisional restorations in the early stages can also improve esthetics and comfort for the patient, with high success rates under favorable occlusal conditions and good primary stability of the implants [7].

Bioactive implant surfaces, due to their capacity to accelerate osseointegration, have been proposed for use in immediate or early loading for implants at posterior and anterior sites in order to further increase the predictability of non-bioactive rough surfaces [8,9].

Kokubo [10] proposed a thermochemical method to obtain titanium implants with a bioactive surface in which titanium and their alloys are first etched chemically with alkaline solutions with high concentration and then submitted to heat treatment at temperature between 600 and 800 °C. The objective of this thermochemical treatment is to reproduce the in vivo formation of crystalline apatite, with the same inorganic composition of the human bone, on the titanium surface, therefore accelerating bone healing and osseointegration [11,12,13].

The chemical treatment, as described by the author, consists of soaking the implant in a 10 M NaOH aqueous solution at 60 °C for 24 h and then gently washing it with distilled water. The thermal procedure consists of implants being heated in an electrical furnace to various temperatures below 800 °C at a rate of 5 °C/min, kept at the temperature for 1 h, and allowed to cool to room temperature in the furnace.

Titanium and titanium alloys are covered with a thin TiO_2_ (titanium oxide) passive layer, and this passivation film is produced spontaneously, which provides high corrosion resistance, lower titanium ion release and durability. During the soaking phase of the chemical treatment, the TiO_2_ layer comes into contact and reacts with the NaOH (sodium hydroxide) solution, forming a hydrated TiO_2_ gel that can be stabilized as an amorphous sodium titanate using a suitable heat treatment [14,15].

**Figure 1:**
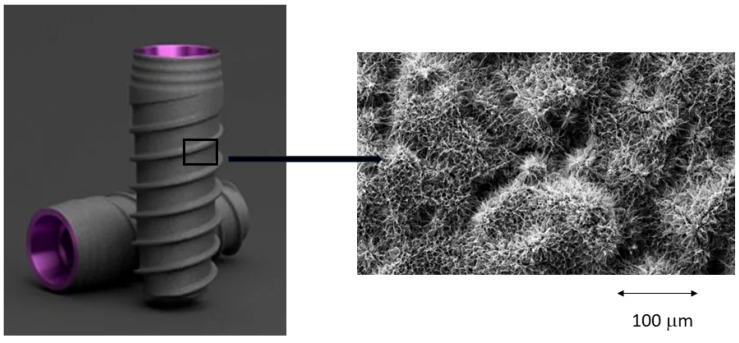
Showing the bioactive surface using Scanning Electron Microscopy, of the implants VEGA (Klockner, Esdaldes-Engorday, Andorra).

The sodium titanate layer is expected to form many TiOH^−^ groups on its surface in the living body via the exchange of surface Na^+^ ions with H_3_O^+^ ions in the surrounding body fluid. These TiOH^−^ groups produce a highly negatively charged surface that initially combines with positive Ca^2+^ ions from human plasma to form amorphous calcium titanate in the surface environment, which later combines with the negative phosphate ions to form amorphous calcium phosphate, transforming into bone-like apatite [12]. This method can be said to provide a biomimetic surface, without the need for osteoblast participation. The surface of the biomimetic dental implants can be observed in Figure 1.

Once the hydroxyapatite layer on the implant surface has formed, the osseointegration process continues with the selective adsorption of fibronectin from human plasma followed by the migration, adhesion, proliferation, and differentiation of osteoblasts, which initiates bone apposition on the surface [12,14,15].

The objective of this study is to determine the growth of sodium titanate crystals in relation to the cooling rate and different calcium contents of concentrated NaOH solutions and, additionally, to analyze the influence of crystal size on the behavior of osteoblasts to achieve the rapid bone mineralization of biomimetic dental implants. The clinical relevance of this research is to provide clinicians with implant surfaces with a hydrophilic microstructure and small crystals to facilitate osteoblast adhesion and trigger rapid mineralization for bone tissue formation.

## 2. Materials and Methods

### 2.1. Materials

**Figure 2:**
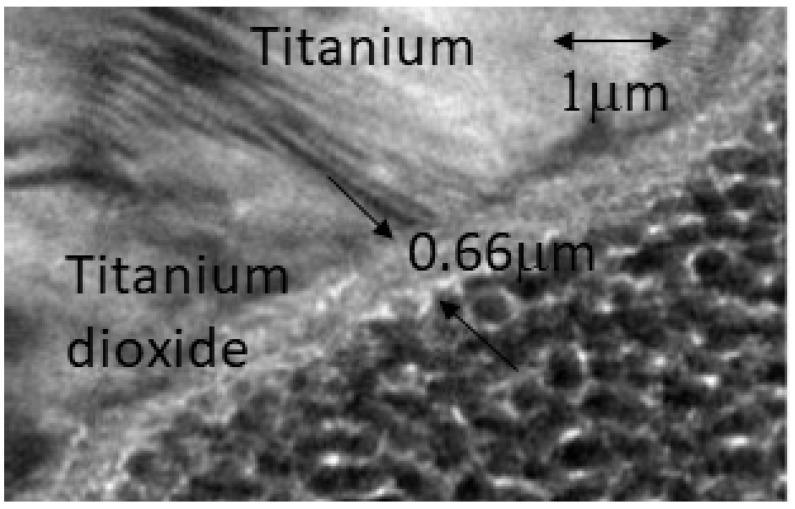
Titanium dioxide obtained by the passivation process.

Three hundred eighty-five discs of cpTi (commercially pure titanium) grade 3 with a diameter of 10 mm were donated by Klockner Dental Implants (Soadco, Escaldes Engordany, Andorra) for this study. The microstructure comprised alpha grains with an average diameter of 50 μm. The titanium was subjected to a passivation process with 40% citric acid for 5 min before the thermochemical treatments. It was washed with distilled water and dried with hot air flow. The average thickness of the passivation layer formed by titanium oxide was determined using a high-resolution Field Emission Scanning Electron Microscope FE-SEM 230( FEI, Hillsboro, OR, USA). In Figure 2, the passivation film on the titanium surface can be observed. The average thickness of the titanium oxide layer was 0.60 ± 0.13 μm. Layer thickness measurements were performed on five titanium disks at 5 different locations for each disk.

### 2.2. Heat Treatments

Twenty samples were used as a control. The samples were only passivated with citric acid.

The thermochemical treatment was conducted according to Kokubo et al. [10]:

- The samples were introduced into a vial containing 10 mL of NaOH 6 M and then placed in an oven at 60 °C for 24 h.
- The samples were carefully rinsed with distilled water and dried in an oven at 40 °C for 24 h.
- The samples were subjected to thermal treatment in a tubular furnace with a 5 °C/min heating rate up to 600 °C and maintained at this temperature for 1 h.
- Eighty samples were cooled to room temperature inside the furnace at different rates: 20 °C/h, 50 °C/h, 75 °C/h, and 115 °C/h (with 20 samples for each cooling rate). The cooling rates were chosen from the lowest rate of 20 °C/h, which corresponds to cooling inside the oven, to 150 °C/h, which corresponds to cooling with the oven door open. The other two speeds are average values in order to determine the influence of the cooling rate on the size of the crystals. The control samples did not undergo any treatment.

**Figure 3:**
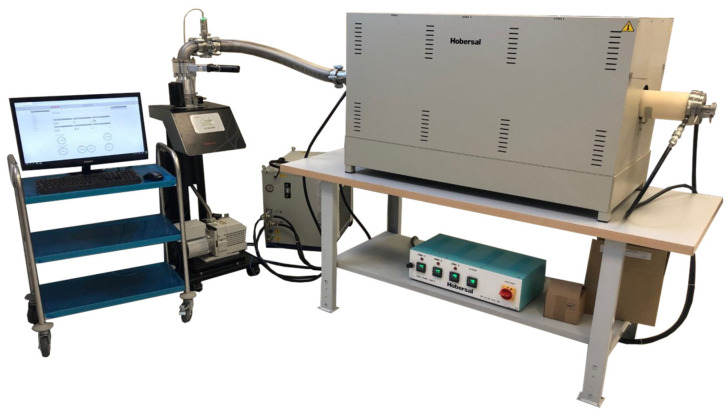
Tubular furnace used for the thermochemical treatment with heating and cooling rate control.

These discs were heat-treated at a predetermined temperature with a tubular furnace (Hobersal ST16, Caldes de Montbui, Spain) with an atmosphere controlled using an Argon 99.99% flow to avoid the oxidation of titanium (Figure 3).

### 2.3. Electron Microscopy

The samples were observed using a TESCAN (CLARA UHR SEM. Brno, Czech Republic) high-resolution scanning electron microscope using a 15 KV electron acceleration at different viewing distances. The microscope has an image analysis system (Image Jversion 1.54f) with a resolution of 2 nm. This equipment was used to determine the average size of the sodium titanate crystals produced at different cooling rates. In addition, the microscope has a system of microanalysis EDX (Oxford Aztec EDX, Oxford, UK).

### 2.4. Sodium Cations Determination

The amounts of sodium cations in the different treatments were studied. For this purpose, 5 samples of the control and of each thermo-chemical treatment were immersed in distilled water with agitation. It is well known that sodium will be totally solubilized in the aqueous medium. The different solutions were analyzed in an Inductively Coupled Plasma (ICP-MS) to determine the sodium ion values after 1 h of immersion at room temperature.

### 2.5. Roughness

White light interferometry (Wyko NT1100 Interferometer, Veeco Instruments, Plainview, NY, USA), in its vertical scanning interferometry mode, was used to produce, evaluate, and quantify topographical features of the tested surfaces. The interferometric technique is ideal for imaging these surfaces as a large area of the surface can be imaged with a high vertical resolution (≈2 nm). The analysis area was 124.4 × 94.6 µm. Data analysis was performed with Wyko 32 (Veeco Instruments, Plainview, NY, USA), which allows the application of a Gaussian filter to separate waviness and form from roughness. Five different specimens of each treatment and control were measured to determine the amplitude parameter (Sa), the maximum peak value (Sz), and the hybrid parameter (Index area) [16,17].

### 2.6. Wettability and Surface Energy

The contact angle analysis was performed on n = 5 samples with ultrapure distilled water (Millipore Milli-Q, Merck Millipore Corporation, Darmstadt, Germany) and formamide (Contact Angle System OCA15plus-Dataphysics, Filderstadt, Germany), and the corresponding data were analyzed with the SCA20 goniometer (Dataphysics, Filderstadt, Germany). Contact angle measurements were made using the sessile drop method. Drops were generated with a micrometric syringe and were deposited over discs. A total of 3 μL of distilled water and 1 μL of formamide were deposited on each sample at 200 μL/min. Finally, the surface free energy was determined by applying the Owens, Wendt, Rabel, and Kaelble (OWRK) equation with wettability values obtained with distilled water and formamide and the Wenzel equation for the correction of contact angles with the roughness [18,19,20].

### 2.7. Osteoblast Study

For in vitro studies, human osteoblast cells (Saos-2; ATCC, Manassas, VA, USA) were cultured in McCoy’s modified 5A medium, supplemented with 10% fetal bovine serum (FBS, Gibco, New York, NY, USA), 1% penicillin/streptomycin 2 mM (Invitrogen, Carlsbad, CA, USA), and 1% sodium pyruvate (Invitrogen, Carlsbad, CA, USA). Cultures were grown at 37 °C in a 5% CO_2_ incubator under humidified conditions, with n = 25 for control and each cooling rate studied.

Confluent cells were incubated with TrypLE (Invitrogen, Carlsbad, CA, USA) for 1 min to detach them from the flask. Subsequently, 5000 cells were seeded on each disc and incubated at 37 °C. After 3 and 14 days of incubation, the samples were washed with PBS and moved onto a new plate to perform a metabolic activity assay using Alamar Blue (Invitrogen-Thermo Fisher Scientific, Waltham, MA, USA), following the manufacturer’s protocol. Briefly, the reagent was prepared and pipetted to cover the samples, and the percentage of Alamar Blue reduction was estimated after 4 h of incubation at 37 °C, using the Alamar Blue solution as a blank.

To study the osteoblasts’ differentiation, the alkaline phosphatase (ALP) activity was determined using the Sensolyte pNPP alkaline phosphatase colorimetric assay (Anaspec, Fremont, CA, USA). The determination of ALP was performed at a wavelength of 495 nm, and detection was carried out using a conventional ELx800 microplate reader (Bio-Tek Instruments Inc., Winooski, VT, USA).

### 2.8. Influence of Calcium Content

Additionally, NaOH 6 M solutions were made, as indicated by Kokubo’s method, with the presence of calcium at two concentrations, one of approximately 1000 ppm and another of 100 ppm. The treatments to obtain sodium titanate were carried out for the samples cooled at 20 °C/h and 75 °C/h with the two solutions, one rich in calcium and the other low in calcium. The crystal size and microstructure were analyzed in order to determine whether high levels of calcium could affect the microstructure. For this purpose, 80 disks were used for the two cooling rates and the two calcium contents.

### 2.9. Statistic Study and Number of Samples

The number of samples used was obtained using an experimental sample size method. Statistical analysis was performed using the MiniTab version 17 software (Minitab Inc., Lock Haven, PA, USA). The Kruskal–Wallis and Mann–Whitney U non-parametric tests were used to compare the different conditions. Statistical differences were considered at *p* < 0.05.

The number of samples was as follows: 5 samples passivation film + 20 samples SEM × 5 (1 control + 4 cooling rates) + 5 samples roughness × 5 (1 control + 4 cooling rates) + 5 samples wettability × 5 (1 control + 4 cooling rates) + 5 samples for Na content × 5 (1 control + 4 cooling rates) + 25 samples cellular studies × 5 (1 control + 4 cooling rates) + 20 samples Ca study × 2 calcium contents × 2 cooling rates = 385 samples.

## 3. Results

**Figure 4:**
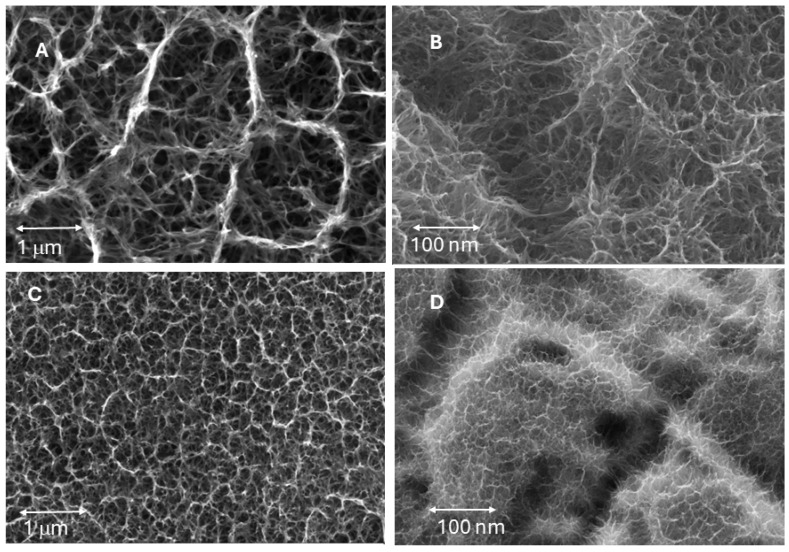

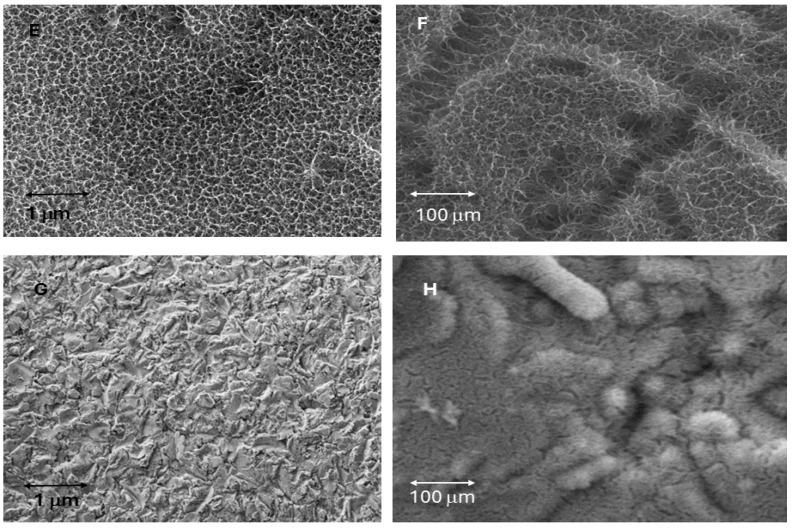
(**A**) Surface of biomimetic dental implant with cooling at 20 °C/h. (**B**) A higher magnification. (**C**) Surface of biomimetic dental implant with cooling at 50 °C/h. (**D**) A higher magnification. (**E**) Surface of biomimetic dental implant with cooling at 75 °C/h. (**F**) A higher magnification. (**G**) Surface of biomimetic dental implant with cooling at 115 °C/h. (**H**) A higher magnification.

Figure 4 shows the surface of the biomimetic dental implant at different cooling rates. It can be seen that as the cooling rate increases, the crystal size decreases. At the fastest speed (115 °C/h), the surface does not show clear crystallization but rather an amorphous phase, although some very fine crystals can be seen in some areas.

The sodium titanate that forms on the surface of the titanium is generated by the chemical reaction with concentrated NaOH and causes a sodium titanate structure in the form of crystals that will grow with temperature. There is no increase in the thickness of the titanium oxide layer as it is the reaction with the titanium oxide that changes the morphology of the crystals formed and gives this crystalline structure. The topography changes due to the cooling rate, which shows that it is a solid-state diffusion effect, just as it occurs in other processes such as grain size growth in metallic materials or in calcium phosphate granules, which depends on the temperature of the materials, time and solid-state diffusion coefficients [21,22,23].

**Figure 5:**
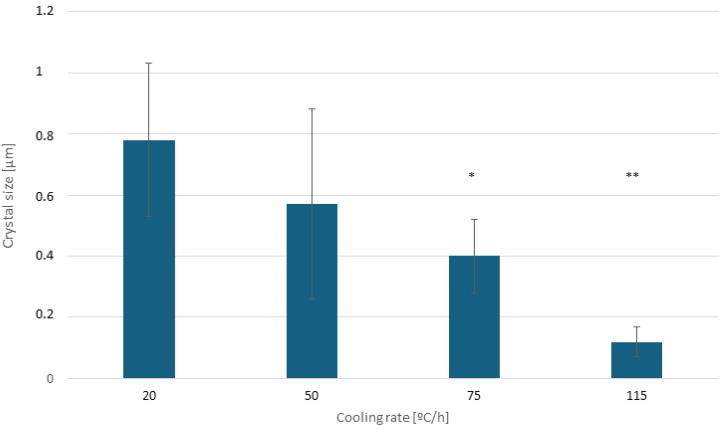
Crystal size average diameter (μm) in relation to cooling rate. One asterisk means statistically significant differences at *p* < 0.05 with the samples without asterisk and with two asterisks. Besides, the samples without asterisk and two asterisks show also statistically significant differences at *p* < 0.05.

Figure 5 shows the average crystal sizes obtained using high-resolution microscopic observation in more than 10 zones per disk.

**Figure 6:**
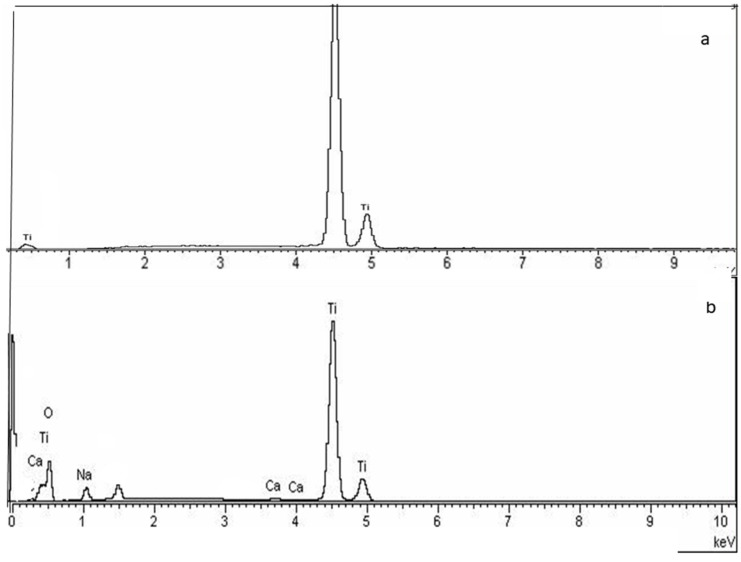
EDX spectra of the samples: (**a**) control and (**b**) sample treated thermochemically at 20 °C/h.

The EDX results of the control sample and the thermochemically treated samples are shown in Figure 6 where the oxygen and sodium peaks produced by the reaction with NaOH can be seen. Between the different cooling rates, no difference between the EDX spectra can be observed.

Table 1 shows the roughness, wettability and the total surface free energy considering the dispersive and polar component of the different surfaces.

The values in Table 1 show that the different cooling rate does not affect the roughness between the different cooling rates or with respect to the control. It can be confirmed that the titanate layer reproduces the original topography of the samples. In none of the different types of treatment were statistically significant differences observed in any of the roughness parameters studied.

The wettability behavior shows that as we increase the cooling rate, a greater hydrophilicity of the surfaces is produced, showing statistically significant differences between them in all cases. The samples with a cooling rate of 115 °C/h show a wettability without statistically significant differences with respect to the control. In the same way, it can be observed in the calculation of surface energies that the structure obtained with a speed of 75 °C/h presents a higher surface energy with statistically significant differences with respect to the other cooling speeds. The speeds of 20 and 50 °C/h also show higher hydrophilicity than the control and those of the 115 °C/h speed. It should be noted that in the surface energy, which is a sum of the dispersive and polar component, the polar component of the samples cooled at the 75 °C/h rate plays the most important role.

Studies of sodium cation content on the surfaces of the different treatments have shown that there are no statistically significant differences between the different thermochemical treatments studied. The differences are clearly with respect to the control, since these discs have not been subjected to the reaction with concentrated NaOH. The sodium contents in this control are due to the impurities that are present in its surface; therefore, the cooling rate does not affect the sodium contents of the samples as can be observed in Table 2.

**Figure 7:**
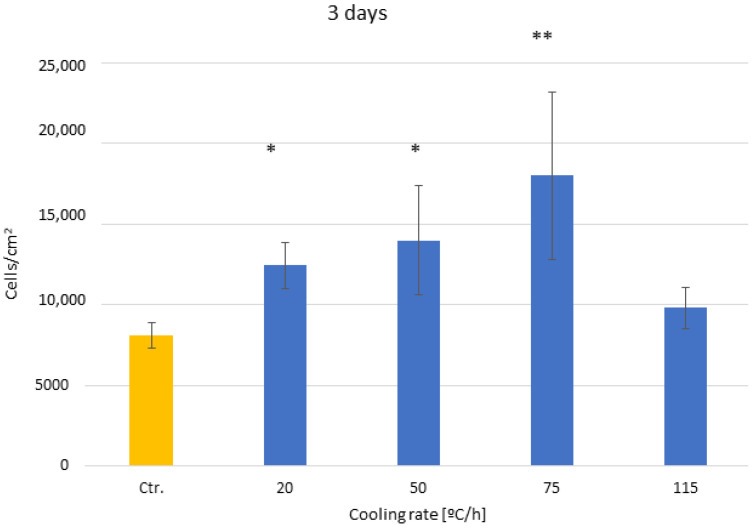
Osteoblast adhesion for the different cooling rates at three days after culture. The different number of asterisks means a statistically significant difference at *p* < 0.05 among them.

Figure 7 shows that in the human osteoblast cultures, the values of cell adhesion at three days are at their maximum for dental implants with smaller crystal sizes except for the culture at the maximum velocity since it has a predominance of the amorphous phase, with values similar to the control. It can also be seen that as the crystal size decreases, osteoblastic adhesion increases. These values show statistically significant differences at *p* < 0.05 between the 20, 50, and 75 °C/h conditions and the rest but not between the control and the conditions at 115 °C/h. It can also be observed that the sizes of the control crystals and those generated at 115 °C/h do not present statistically significant differences since, as can be seen in the microstructure, there is no complete crystallization under this cooling rate.

**Figure 8:**
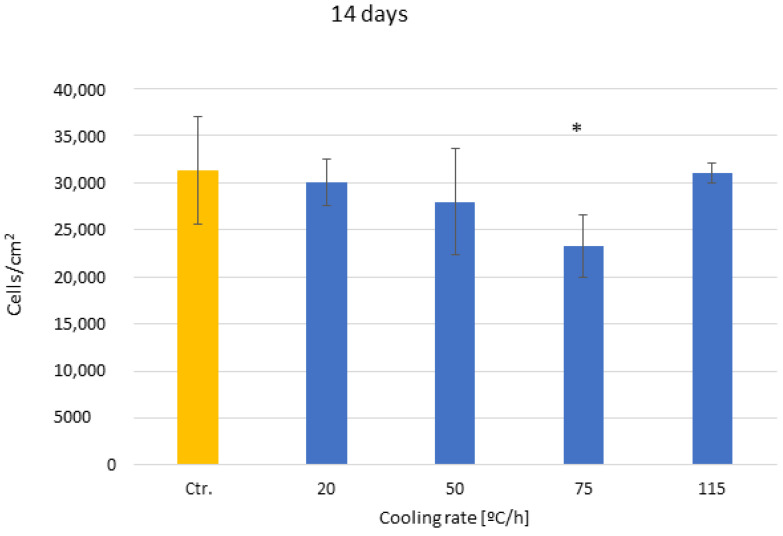
Osteoblast adhesion for the different cooling rates at fourteen days after culture. The different number of asterisk means a statistically significant difference at *p* < 0.05 among them.

The results of cell adhesion at 14 days show a change in trend, where the samples cooled at a speed of 75 °C/h present lower adhesion than the others, with statistically significant differences. This is because adhesion to the surface has practically completed, and the cells are in the proliferation and mineralization phase, as can be seen in Figure 8. The other speeds do not present statistically significant differences.

**Figure 9:**
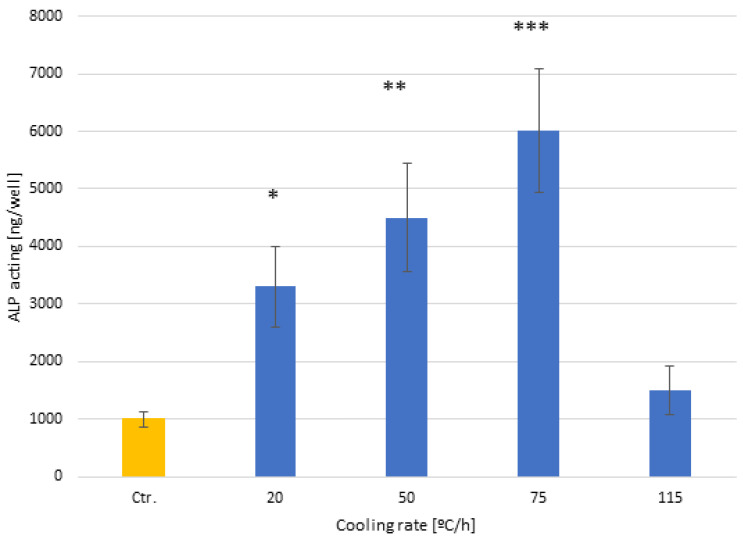
Alkaline phosphatase activity after 14 days for the different cooling rates. The different number of asterisks means a statistically significant difference at *p* < 0.05 among them.

This result is corroborated in Figure 9, in which the degree of mineralization at 14 days reaches a maximum for the samples cooled at a speed of 75. The alkaline phosphatase activity shows growth as the cooling speed increases, showing statistically significant differences at all speeds. The control surfaces do not show statistically significant differences from the surfaces cooled at a speed of 115 °C/h, which do not have a crystalline surface as we have seen.

**Figure 10:**
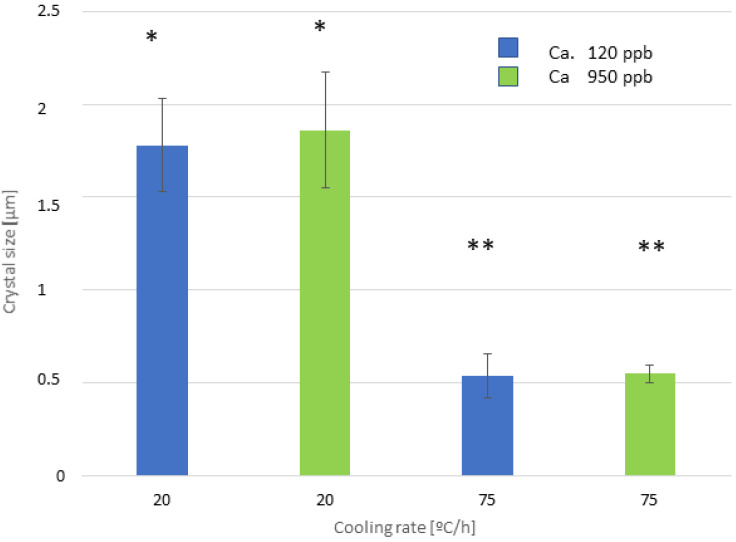
Average crystal size using different calcium contents in the solution and different cooling rates. The different number of asterisks means a statistically significant difference at *p* < 0.05 among them.

**Figure 11:**
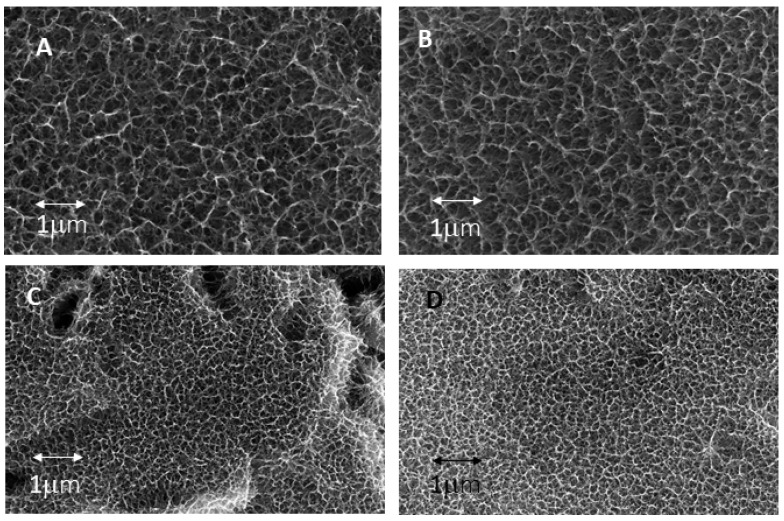
(**A**) Microstructure obtained with a cooling rate of 20 °C/h and 950 ppb of calcium in the solution. (**B**) Microstructure obtained with a cooling rate of 20 °C/hand 120 ppb of calcium in the solution. (**C**) Microstructure obtained with a cooling rate of 75 °C/h and 950 ppb of calcium in the solution. (**D**) Microstructure obtained with a cooling rate of 75 °C/h and 120 ppb of calcium in the solution.

The influence of calcium on the growth of sodium titanate crystals has been determined, and no statistically significant differences in crystal size were observed. The results can be seen in Figure 10, and the microstructures for the two calcium contents are shown in Figure 11. The different commercial NaOH solutions have different purities ranging, in general, from 100 ppb of calcium to 1000 ppb. We wanted to determine if the oscillations of the calcium concentrations in the NaOH solutions could vary the measurements in the sizes of the crystals as well as their biological activity [24,25].

It can be seen from the crystal sizes and morphologies obtained by SEM that there is no statistically significant influence on the values of crystal size and morphology as a function of the calcium contents studied.

**Figure 12:**
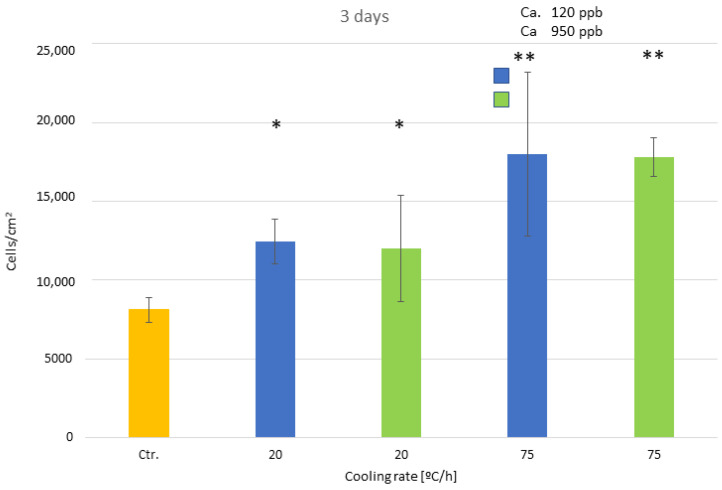
Osteoblast adhesion for the different cooling rates and calcium content at three days after culture. The different number of asterisks means a statistically significant difference at *p* < 0.05 among them.

**Figure 13:**
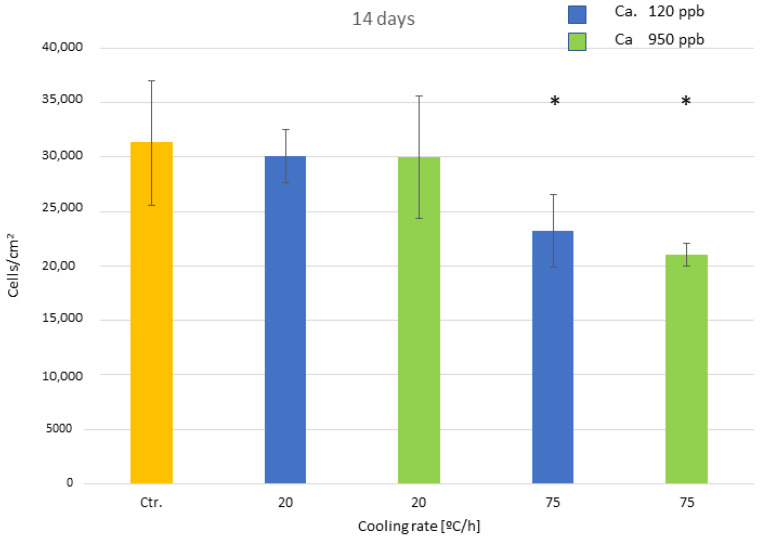
Osteoblast adhesion for the different cooling rates and calcium content at fourteen days after culture. The different number of asterisk means a statistically significant difference at *p* < 0.05 among them.

Figure 12 shows the number of osteoblastic cells at 3 days and Figure 13 at 14 days. It can also be seen that the influence of the calcium content up to 950 ppb does not influence osteoblast activity and follows the same trend as the previous results of NaOH without calcium cations.

**Figure 14:**
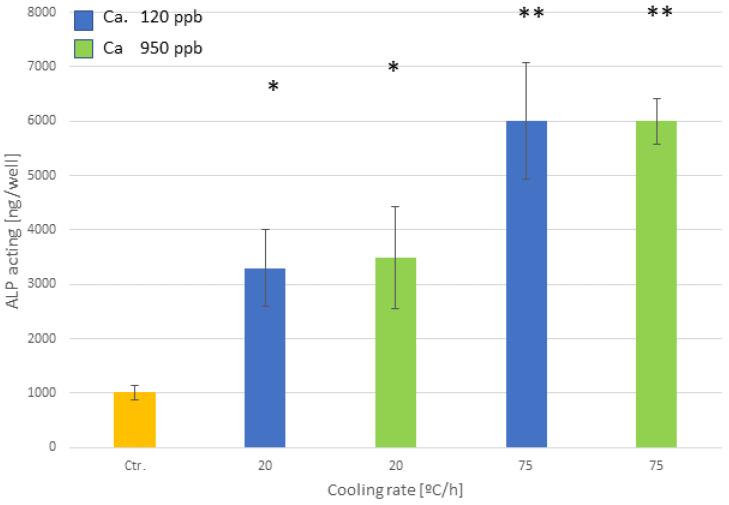
Alkaline phosphatase activity after 14 days for the different cooling rates and different calcium contents. The different number of asterisks means a statistically significant difference at *p* < 0.05 among them.

The results of alkaline phosphatase show no statistically significant differences *p* < 0.05 with the different calcium contents, but there is an influence with the cooling rate, as can be observed in Figure 14.

## 4. Discussion

From the results of the crystal sizes obtained at different cooling rates in titanium processing, it is possible to grade the biological behavior of the osteoblastic cells. It is well known, in material science, that fast cooling rates produce more crystallization nuclei of the material to be processed, i.e., there are more nuclei due to the undercooling that occurs. When the cooling rate is slow, the degree of subcooling is smaller, and, therefore, the numbers of crystal nuclei are reduced [26,27,28]. When there are many nuclei, the crystallization rate is faster, and we are able to obtain structures of many crystals and of a smaller size than those cooled slowly. This behavior can also be seen in materials based on calcium phosphates, where when the particles are smaller, the material is more reactive than that of larger particles [19,20,21,22,23,24,25,26,27,28,29,30]. As shown by Geneva et al., small particles are more reactive than large particles due to an increase in the specific surface area able to react with the medium to form apatites [31,32,33]. For this reason, bone regeneration materials can be found that present small calcium phosphate granules for rapid bone formation or large granules for slower regeneration rates.

When the cooling speeds are very fast, as in the case of 115 °C/h, there is not enough time for the formation of clusters or crystallization nuclei, and an amorphous material is obtained on a part of the surface dotted with very small crystals. The amorphous part is not so favorable for osteoblast adhesion, and, as we have seen, it is very similar to the untreated surface.

From the roughness results obtained, it can be affirmed that the thermochemical treatment does not affect the original roughness of the samples. The titanate layers that are formed reproduce the topography, and the appreciating values of Sa, Sz and area index are very similar with no statistically significant differences in any case. Therefore, the roughness of the samples will not be a factor affecting osteoblastic cell activity. However, we have been able to observe the improvement of the hydrophilicity in the samples cooled at a speed of 75 °C/h, statistically superior to the hydrophilicity of the titanate layers obtained at speeds of 20 and 50 °C/h. The increase in hydrophilicity will allow a greater adsorption of proteins in the physiological media and therefore favors the adhesion of osteoblasts than in the other cases. We can also say that, in all cases, the presence of titanate crystals improves hydrophilicity and produces an increase in surface energy with respect to the control and the higher speed, which forms an amorphous structure [34,35]. It should be noted that the polar component grows more with respect to the dispersive component in the calculation of the surface energy. As is well known, this increase in the polar component favors osteoblast adhesion [36,37,38].

It has been possible to verify how the adhesion of osteoblastic cells, after three days, is higher for the treatment at a speed of 75 °C/h, when compared to the other cooling speeds. Increasing cell adhesion behavior can be seen as the cooling rate increases, since there are more and smaller crystals, significantly increasing the specific surface area, i.e., the surface area susceptible to cell adhesion [36,37,38,39,40,41]. It only decreases with the highest speed analyzed (115 °C/h), as we have mentioned, due to the difficulty of crystalline growth. On the other hand, it should be noted that this cell adhesion decreases for the case of 75 °C/h at 14 days, and this is due to the fact that the cells are already in the process of differentiation, also called mineralization, as indicated by the significant levels of alkaline phosphatase found [42,43,44,45]. In other words, the cooling rate of 75 °C/h will be the surface that will most accelerate the mineralization process in a dental implant. The clinical implication of this result is that we will be able to have dental implants with faster bone formation speeds in the clinic, which will allow the clinician to reduce the time to load the dental implant with the prostheses, and the patient will have all his or her functionality and esthetics recovered in less time [46,47,48,49,50,51,52].

This biomimetic surface of different crystal sizes varies the speed of bone mineralization but not in the bone implant contact at the end of the process of bone growth. That is, the 75 °C/h implant will mineralize faster, but over time, the degree of bone growth between the control and this 75 °C/h implant will be the same [53]. It seems that optimizing the cooling rate and, consequently, mineralization will be important for immediately loaded or early loaded implants that some clinicians prefer for their patients [54,55].

Another important result is that the calcium levels studied do not affect the crystal sizes or even the microstructure of the material. Kokubo et al. [10] indicated that high levels of calcium could negatively influence the formation of a biomimetic crystal structure, since calcium could substitute for sodium and inhibit bioactivity, as the biomimetic structure would not form [56,57]. However, the studied levels corresponding to the possible calcium contents present in NaOH solutions do not affect the properties of the biomimetic sodium titanate surface. The calcium contents studied also do not show variations in the osteoblastic adhesion activity at 3 and 14 days, nor in the mineralization behavior. No statistically significant differences were observed with a *p* < 0.005 between the calcium contents studied. However, as in the case of the solutions without calcium, the different cooling rates studied cause the differences in the crystal sizes and consequently in the cellular activity.

The study of the calcium content in NaOH solutions responds to research by Kokubo et al. [10] which indicated that when calcium cations were present in titanium, a decrease in osteoblastic biological activity occurred. This fact responds to the fact that calcium replaces sodium cations and blocks the formation of apatite in contact with the human physiological environment; therefore, titanium would lose osteoconductive capacity. This research demonstrates that the quantities of calcium present as impurities in concentrated NaOH solutions do not affect the behavior of titanate on the surface of the implants. It was found that the size of titanate crystals, osteoblast adhesion and alkaline phosphate levels do not present statistically significant differences with respect to the alkaline solutions used without the presence of calcium ions.

In this research, it has been possible to determine that the cooling rate causes a decrease in the size of the titanate crystals that will favor the rapidity in the osteoblastic biological activity as the size decreases. The decrease in crystal size has a limit of about 150 °C/h due to the fact that titanium does not crystallize. We have carried out these tests with NaOH concentrations of 6 M and other authors have worked with 10 M, but we have been able to verify [14] that with these concentrations, the same type of osteoconductive morphologies can be obtained. Another possible line of future work is that these results can also be transferred to other titanium alloys used as biomedical implants, such as Ti6Al4V, Ti13Zr or low-elastic-modulus titanium-based alloys with the presence of Zr, Nb, and Ta.

The clinical significance of these thermal treatments could be interesting to have more biologically active osteoconductive implants for patients with deficiencies in bone tissue metabolism, such as diabetics [58,59], osteoporotic [60,61], smokers [62,63] or oncological patients [64]. The small crystals could help osteoblastic cell activity, which in these patients is clearly diminished [59,60,61]. These applications have already been obtained for granules based on calcium phosphates that surgeons introduce in bone-deficient sites in order to regenerate the bone. It is well demonstrated that small granules have a much faster bone formation than large granules due to the specific surface of the biomaterial that interacts with proteins and cells [65]. On the other hand, surfaces with very small titanates would be suitable for immediate loading implants as they would facilitate mineralization, and therefore, biological fixation would be obtained at shorter times than with larger titanate crystals obtained at slower speeds.

From the point of view of dental implant manufacturing, it is important to know that faster cooling rates also produce titanate crystalline titanate structures that will favor cellular activity. This fact reduces the manufacturing times and reduces the economic cost of the implants.

This treatment of sodium titanate formation causes it to form apatite when in contact with the blood fluid, thus making the implant osteoconductive. There are other ways to obtain the improvement of osteoblastic activity as shown in the work of Papynov et al. [65], where they present a bioactive material, CaSiO_3_-HAp biocomposite ceramics reinforced with a Ti6Al4V titanium alloy matrix obtained by additive manufacturing. This material is new and represents a prospect for the creation of high-tech implant products for regenerative bone surgery.

The findings of this study should be completed with in vivo test results, which we hope to finish in the near future. In any case, we found that it was possible to optimize the size of the titanate crystals to obtain the most active microstructure for osteoblastic cells by means of the cooling rate. It was also possible to confirm that the presence of calcium below 900 ppm in the solutions does not affect the biomimetic topography.

## 5. Conclusions

It was determined that increasing the cooling rate results in smaller sodium titanate structures that are more hydrophilic and have higher total surface energy than the control ones. The different cooling rates do not affect the roughness or surface sodium concentrations. The smaller crystal size results in higher osteoblastic cell activity and a higher degree of mineralization. It was observed that the speed of 150 °C/h produces an amorphous structure and does not present good biological properties. The optimum cooling rate of those studied was 75 °C/h. It was determined that the calcium impurities that may be present in NaOH solutions do not inhibit the biological activity of osteoblasts. These treatments are encouraging for producing more osteoconductive surfaces that favor the rapid mineralization of bone tissue.

## Figures and Tables

**Table 1:** Roughness parameters Sa, Sz and index area, contact angle (CA), dispersive component of the surface energy (DC), polar component (PC) and total surface energy (SFE). Asterisks mean the statistical difference significance *p* < 0.05.

	Sa (µm)	Sz (µm)	Index Area	CA (°)	DC (mJ/m^2^)	PC (mJ/m^2^)	Total SFE (mJ/m^2^)
Control	0.21 ± 0.02	0.34 ± 0.02	1.09 ± 0.01	77 ± 5	24.8 ± 1.2	10.2 ± 2.0	35.0 ± 3.2
20	0.24 ± 0.10	0.41 ± 0.11	1.08 ± 0.06	58 ± 3 *	27.2 ± 1.2 **	18.3 ± 1.8 *	45.5 ± 2.2 *
50	0.26 ± 0.15	0.67 ± 0.47	1.06 ± 0.04	50 ± 4 **	27.7 ± 1.3 **	17.5 ± 2.1 *	45.2 ± 1.2 *
75	0.22 ± 0.18	0.59 ± 0.67	1.15 ± 0.04	42 ± 2 ***	29.0 ± 2.2 **	20.5 ± 1.9 **	49.5 ± 1.8 **
115	0.25 ± 0.18	0.33 ± 0.17	1.07 ± 0.08	70 ± 7	25.0 ± 1.1	10.5 ± 1.8	35.5 ± 4.2

**Table 2:** Sodium cations determined for different samples studied. One asterisk means statistically significant differences at *p* < 0.05 with the samples without asterisk.

**Samples**	**Concentration Na^+^ (ppm)**
Control	24 ± 5
20	1174 ± 298 *
50	1289 ± 312 *
75	1199 ± 288 *
115	1135 ± 234 *

## Data Availability

Dataset available on request from the authors.
